# Transplantation of human embryonic stem cells alleviates motor dysfunction in AAV2-Htt171-82Q transfected rat model of Huntington’s disease

**DOI:** 10.1186/s13287-021-02653-7

**Published:** 2021-11-22

**Authors:** Jaisan Islam, Kyoung Ha So, Elina KC, Hyeong Cheol Moon, Aryun Kim, Sang Hwan Hyun, Soochong Kim, Young Seok Park

**Affiliations:** 1 Department of Neuroscience, College of Medicine, Chungbuk National University, Cheongju, Republic of Korea; 2 Institute for Stem Cell & Regenerative Medicine (ISCRM), College of Veterinary Medicine, Chungbuk National University, Cheongju, Republic of Korea; 3 Laboratory of Veterinary Embryology and Biotechnology (VETEMBIO), College of Veterinary Medicine, Chungbuk National University, Cheongju, Republic of Korea; 4 Department of Neurosurgery, Gammaknife Icon Center, Chungbuk National University Hospital, Cheongju, Republic of Korea; 5 Department of Neurology, Chungbuk National University Hospital, Cheongju, Republic of Korea; 6 Department of Neurosurgery, Chungbuk National University Hospital, College of Medicine, Chungbuk National University, 776, 1 Sunhwanro, Seowon-gu, Cheongju-si, Chungbuk 28644 Republic of Korea

**Keywords:** Adeno-associated virus, Huntington's disease, Human embryonic stem cell, Nanoparticles, Cell tracking

## Abstract

**Background:**

Human embryonic stem cells (hESCs) transplantation had shown to provide a potential source of cells in neurodegenerative disease studies and lead to behavioral recovery in lentivirus transfected or, toxin-induced Huntington's disease (HD) rodent model. Here, we aimed to observe if transplantation of superparamagnetic iron oxide nanoparticle (SPION)-labeled hESCs could migrate in the neural degenerated area and improve motor dysfunction in an AAV2-Htt171-82Q transfected Huntington rat model.

**Methods:**

All animals were randomly allocated into three groups at first: HD group, sham group, and control group. After six weeks, the animals of the HD group and sham group were again divided into two subgroups depending on animals receiving either ipsilateral or contralateral hESCs transplantation. We performed cylinder test and stepping test every two weeks after AAV2-Htt171-82Q injection and hESCs transplantation. Stem cell tracking was performed once per two weeks using T2 and T2*-weighted images at 4.7 Tesla MRI. We also performed immunohistochemistry and immunofluorescence staining to detect the presence of hESCs markers, huntingtin protein aggregations, and iron in the striatum.

**Results:**

After hESCs transplantation, the Htt virus-injected rats exhibited significant behavioral improvement in behavioral tests. SPION labeled hESCs showed migration with hypointense signal in MRI. The cells were positive with βIII-tubulin, GABA, and DARPP32.

**Conclusion:**

Collectively, our results suggested that hESCs transplantation can be a potential treatment for motor dysfunction of Huntington's disease.

## Background

Huntington’s disease (HD) is an inherited disorder with the progressive loss of brain and muscle function [1, 2]. The motor dysfunction is primarily associated with degeneration of GABAergic medium spiny projection neurons in the basal ganglia, particularly the striatum, due to the expansion of cytosine-adenine-guanine (CAG) repeat leading to a malfunctioned mutant huntingtin protein (mHtt) [3]. As the disease progresses, the severe loss of GABAergic projection neurons in the striatum is followed by the selective degeneration of neurons in other regions such as the substantia nigra and several cortical areas [4, 5]. Though there are still no satisfactory treatment approaches for HD, there are few symptomatic treatment manners regarding chorea-based neurochemical pathology which may have favorable effects on motor function, quality of life, and safety [6, 7].

Various viral-vector mediated gene therapy has been used to make an HD animal model for understanding the pathophysiology of HD and to apply new therapeutic approaches. But due to its low immunogenicity and less pathogenicity combined with the capability of long-lasting transgene expression, the adeno-associated virus has become an ideal option [8]. Several studies are pursued to delay or prevent the onset of HD or slow its progression by considering disease-modifying strategies which could be categorized into three main groups: reducing the level of the mutant Huntingtin protein, improving neuronal survival, and replacing lost neurons [9–13].

Cell transplantation may provide an effective reparative therapeutic approach in this scenario for replacing the neuronal death cells in the striatum and other affected brain regions [14–16]. There are two major categories of cell therapy used in the context of HD. One is using fetal tissues or cells and another one is using stem cells [15]. Compared to fetal cells or tissues, stem cells have been ideal for cell transplantation purposes in HD because they are relatively easy to obtain, and somatic stem cells, in particular, offer a means of eliminating immune rejection problems. Besides, stem cells can self-renew continuously, produce progeny, and differentiate into many cell types including neurons, astrocytes, and oligodendrocytes [15]. Earlier studies showed that embryonic stem cells (ESCs) could differentiate into neural precursors and GABA neurons in the striatum and induce behavioral recovery in the quinolinic acid (QA) animal model of HD [3, 17]. However, there is very little information and evidence about the effect of human ESCs (hESCs) in the HD rat model generated by direct injection of the adeno-associated viral vector carrying the human mutant Htt gene into the striatum.

Magnetic resonance imaging (MRI) is a non-invasive technique to track transplanted cells in longitudinal in vivo studies [18]. This method has been offering stem cell researchers a tool to monitor the cell’s survival, migration, differentiation, and regenerative impact [19]. With the help of using a contrast agent, increasing visibility of transplanted cells and accuracy in image analysis has been achieved through MRI. Among different contrast agents, superparamagnetic iron oxide nanoparticle (SPIONs) is a potential one, which can enhance the contrast between different tissues present by inducing a darker area. SPIONs appear to be the preferred MRI contrast agents for monitoring stem cells due to their high sensitivity and excellent biocompatibility. In recent years, SPION-labeled stem cell detection in MRI has been used in studies for myocardial infarction, stroke, HD, Parkinson’s disease, multiple sclerosis, and amyotrophic lateral sclerosis [20–23].

Therefore, in our study, we investigated the potential of using ultra-high-field MRI to monitor hESCs labeled by SPIONs that transplanted to Huntington’s disease rat brains and whether it causes improvement in motor function of the HD rats after hESCs transplantation.

## Materials and methods

### Experimental subjects

**Fig. 1:**
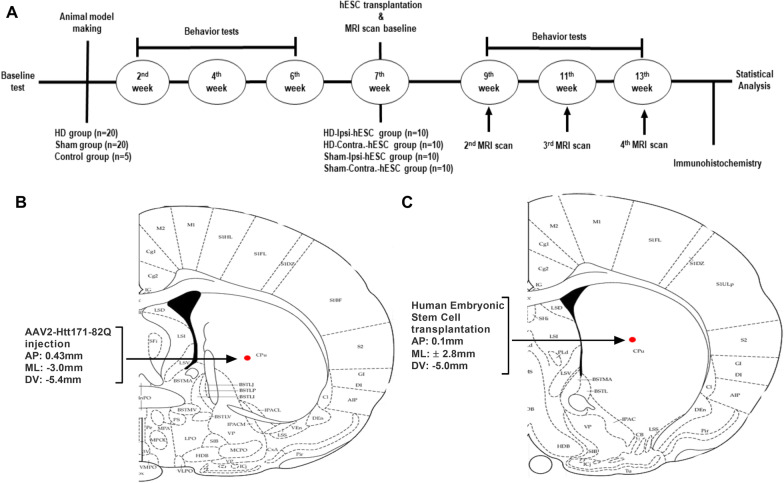
Experimental timeline. **A** The animals were injected with AAV-Htt171-82Q or AAV-GFP into the right striatum and assigned to the HD group (*n* = 20), sham group (*n* = 20), and control group (*n* = 5). Behavioral tests were performed every 2 weeks. The hESCs were transplanted 7 weeks after infection and an MRI scan was conducted for baseline information. SPION cell tracking was managed once per 2 weeks after that and after the third time scanning, the rats were transcardially perfused for histological examinations. **B** Location of AAV-Htt171-82Q or AAV-GFP injection into the right striatum with stereotaxic coordination. **C** Location of hESCs transplantation into the right and left striatum with stereotaxic coordination

The present study included a total of 45 adult female Sprague–Dawley rats (age: 8 weeks; Koatech, Pyeongtaek, Korea), each weighing 200–250 g on arrival. All the animals were maintained at constant temperature (22 ± 1 °C) and humidity (50–60%) with ad libitum access to fresh food and water under a 12:12 h light and dark cycle (light cycle beginning at 8 AM). All tests were conducted in a randomized, double-blind, controlled manner and under the guidelines of the Animal Care and Use Committee (IACUC) of Chungbuk National University (CBNUA-1346-20-02) in Korea. Before the surgery, animals were randomly divided into three groups: HD group (*n* = 20) where the animals were injected with AAV2-Htt171-82Q into the right caudal putamen (CPu), Sham group (*n* = 20) where the animals were injected with AAV2-GFP into the right CPu and control group (*n* = 5). The timeline of the experimental protocol is shown in Fig. 1A.

### Behavior tests

Behavioral tests were performed by an examiner who was unaware of the group of the animals.

#### Stepping test

The stepping test was performed 2 days before the viral vector injection for baseline data and every two weeks interval after viral vector injection and hESCs transplantation. The rats were taken into the behavior test room at first and acclimated there for 30 min. Then, the animals were put on a treadmill for walking by themselves at the speed of 18 cm/s in 10 min followed by a resting period of 5 min before testing. Once, the animals get accustomed to the treadmill, the examiner uplifted both hind limbs and grasped their right forelimb firmly. After that the examiner let them step on the treadmill by their left forelimb only and recorded the number of steps in 10 s. The same procedure was followed with the right forelimb and recorded the number of steps in 10 s [24]. The test was done 3 times for each animal and the mean value was taken for evaluation.

#### Cylinder test

The cylinder test was also accomplished 2 days before the surgery for baseline data and at every two weeks interval after viral vector injection and hESCs transplantation. After the animals got adapted in the test room, the examiner placed the rat inside of a transparent glass cylinder (20 cm diameter, 38 cm height) and counted the times that the rat used its left, right, and both forelimbs to touch the cylinder wall. The total of times counted was set at 20 [24]. The calculation of the asymmetric score was done by using the formula: (targeted forelimb + both/2)/(affected forelimb + non-affected forelimb + both).

### Viral vector

#### Viral vector construction

We customized pAAV2-CMV-HTT171-82Q-IRES-YFP-WPRE (viral titer 1 × 10^13^ GC particles/ml) from the SIRION Biotech company (SIRION, Martinsried, German). By using a restriction enzyme, the human huntingtin cDNA encoding for the first 171 amino acids of the protein along with 82 CAG repeats was cloned into transfer vectors with a cytomegalovirus (CMV) internal promoter. A mixture of 1:1 of the two packaging plasmids (pDP1rs; Plasmid Factory; Cat#:PF401 and pDP2rs; Plasmid Factory; Cat#: PF402) was used to produce a hybrid serotype called AAV1/2 and AAV production cell line was HEK 293T. Viral particles were concentrated and formulated using Vivaspin 20 Centrifugal Concentrator. Iodixanol gradient ultracentrifugation was used for purification and virus was dissolved in PBS + 0.014% Tween 20. Titration of viral particles was performed by qPCR titer method and two PCR primers (ITR_qPCR_F CGGCCTCAGTGAGCGA and ITR_qPCR_R GGAACCCCTAGTGATGGAGTT) were used. ATCC standard reference standard was used and included in all PCR runs as internal calibrator of the viral titers determined. We used CMV because it is the most variable, a strong promoter that is able to produce enough protein for further analysis and the CMV promoter regulates more stable expression of the gene in AAV vectors. Moreover, it has also been observed that CMV can transduce oligodendrocytes, astrocytes in vivo.

#### Viral vector injection to the rat brain

Surgical procedures were performed under general anesthesia. At first, the rats were given some moments to get adapted to the environment and subsequently injected intraperitoneally (i.p.) a mixture of 15 mg/kg Zoletil and 9 mg/kg Rompun in saline and then mounted onto the surgical field. Each rat was injected with 2 µl AAV2-Htt171-82Q or AAV2-GFP into the right caudal putamen at the following coordinates: AP − 0.43 mm, ML − 3.0 mm relative to bregma, and DV − 5.4 mm from the dura (Fig. 1B) as the loss of medium spiny neurons and degeneration of cells starts in caudate and putamen in HD [25]. The flow rate was 0.2 µl/min, and the injection was performed using a 10 µl Hamilton syringe and an automatic microsyringe pump. After injection, the needle was kept in place for 5 min and then slowly retracted.

### hESCs transplantation

#### hESCs preparation

Parthenogenetic hESCs were obtained from the Wisconsin International Stem Cell Bank (WiCell Research Institute, Madison, WI, USA). hESCs cell line information is the following, product name: ESC WA09, alias: H9, lot number: WB0143, parent material: WA09-MCB-01, passage number: p26. Purchased hESCs were transitioned to feeder-independent condition by the provider and cultured according to the protocol from Wicell Research Institute. Cells were maintained on Matrigel-coated plates in mTeSR1 (Stemcell Technologies) with daily medium change. All cells were grown in colonies at 37 °C and 5% CO2 and cells were passaged every 4 to 6 days with 1 mg/ml Dispase (Stemcell Technologies). The only colony-type culture was used during regular propagation and throughout the experiment [26, 27]. We used parthenogenetic ESCs because it has been found that they have chances of formation of smaller teratoma than normal ESCs [28].

#### Cells transplantation

After six weeks of Htt virus injection, all the animals of the HD group were divided randomly into two sub-groups: HD-ipsi-hESC (*n* = 10) and HD-contra-hESC (*n* = 10). In the HD-ipsi-hESC group, hESCs were transplanted into the ipsilateral side of AAV2-Htt171-82Q injection (right CPu) and in the HD-contra-hESC group, hESCs were transplanted into the contralateral side of AAV2-Htt171-82Q injection (left CPu). Sham grouped animals were also separated into two sub-groups in a similar way to HD animals: Sham-ipsi-hESC (*n* = 10) and Sham-contra-hESC (*n* = 10). Then, stem cells were transplanted into the striatum (right CPu) of HD-ipsi and Sham-ipsi group animals at the following coordination: AP 0.1 mm, ML 2.8 mm relative to the bregma, DV − 5.0 mm from the dura (Fig. 1C). In the HD-contra. and Sham-contra. group animals, stem cells were transplanted into the striatum (left CPu) at the following coordination: AP 0.1 mm, ML − 2.8 mm relative to the bregma, DV − 5.0 mm from the dura. The volume of hESC was 5 μl per rat (1.25 × 10^5^ cells/μl). The transplantation procedure was done with the same techniques as the injection of the viral vector.

### In vivo MRI scanning

MRI scan was conducted in five randomly-chosen animals from each HD-ipsi-hESC, HD-contra-hESC, Sham-ipsi-hESC, and Sham-contra-hESC group. Baseline data were acquired before the stem cell transplantation on the 7th week. After that, at an interval of every two weeks (9th week, 11th week, and 13th week) 2nd, 3rd, and 4th MRI scans were performed respectively to monitor the migration of SPION-labeled ESCs. MR images were acquired at ultra-high field 4.7 Tesla Bruker Biospec Scanner (KBSI, Ochang, Korea). We conducted high-resolution T2 weighted sequences (repetition time (TR) = 5000 ms, echo time (TE) = 20 ms, flip angle = 90°, field of view (FOV) = 40 × 40 mm^2^, matrix = 256 × 256, section thickness = 1 mm) and T2* weighted sequences (TR = 500 ms, TE = 12 ms, flip angle = 30°, FOV = 40 × 40 mm^2^, matrix = 256 × 256, section thickness = 1 mm) in the coronal plane of the rat head. The T2 and T2* images were analyzed by RadiAnt DICOM Viewer software (https://www.radiantviewer.com) and the stem cells were characterized as the hyperintense signal area in T2 images and hypointense signal area in T2* images [23].

### Histology examinations

After MRI scanning and behavior test at week 13th, the rats were deeply anesthetized with a mixture of 15 mg/kg Zoletil and 9 mg/kg Rompun in saline. Then the rats were transcardially perfused with phosphate-buffered saline (PBS), followed by 4% paraformaldehyde. The brains were extracted and fixed overnight in the same postfixed solution, followed by dehydration in 30% sucrose.

For immunostaining, the rat's brains were dehydrated in a series of ethanol and embedded in paraffin. Coronal sections of the striatum were cut by a cryostat 10 µm thin and mounted on slides. After drying at room temperature overnight, the sections were deparaffinized and hydrated to distilled water. The sections then were incubated with serum blocking solution for 1 h and with mouse anti-HTT protein (1:200, clone mEM48, Merck, Darmstadt, Germany), mouse anti-ionized calcium-binding adapter molecule 1 (Iba-1) (1:200, Abcam, Cambridge, MA, USA), mouse anti-glial fibrillary acidic protein (GFAP) (1:200, Abcam), rabbit anti-oligodendrocyte transcription factor (Olig2) (1:200, Abcam), goat anti-NeuN (1:200, Abcam), and rabbit anti-cleaved caspase 3 (1:200, Abcam) antibodies overnight. The corresponding secondary antibody was applied before the sections were stained with DAB (Vector Laboratory, California, USA). Nuclei were counterstained with hematoxylin. Semi-quantification (a method for investigating protein expression and localization within stained tissues [29]) of different antibody-positive cells was performed by the ImageJ software (National Institutes of Health, Bethesda, MD, USA) with the following formula:

“antibody-positive cells (%) = (number of cells stained with antibody/total number of cells per field) × 100.

To observe the presence of iron, the sections were immersed in the mixed solution of hydrochloric acid and potassium ferrocyanide for 20 min and counterstained with nuclear fast red for 5 min. Finally, the sections were dehydrated and mounted with coverslips. To investigate the differentiation of injected stem cells, we stained the sections with anti-β-Tubulin III antibody (1:200, T8578, Merck), anti-GABA antibody (1:200, A2052, Merck), and anti DARPP32 antibody (1:200, ab40801, Abcam). Frozen sections were incubated with these primary antibodies at 4 °C overnight followings by incubating with Alexa Fluor® 488 (1: 500, ab150077 and ab150113, Abcam). The sections were counterstained with DAPI.

### Statistical analysis

Data were analyzed using GraphPad Prism (GraphPad Software version 8.4.2, Inc., San Diego, CA, USA) and represented as the mean ± standard deviation for the behavior tests. In all behavioral result analyses, a comparison of mean values was conducted with a one-way ANOVA test followed by Tukey's multiple comparison test. A p-value of < 0.05 was considered statistically significant. In the semi-quantification analysis of histology slides, ordinary one-way analysis of variance was used.

## Results

### Motor dysfunctions after HD model making

**Fig. 2:**
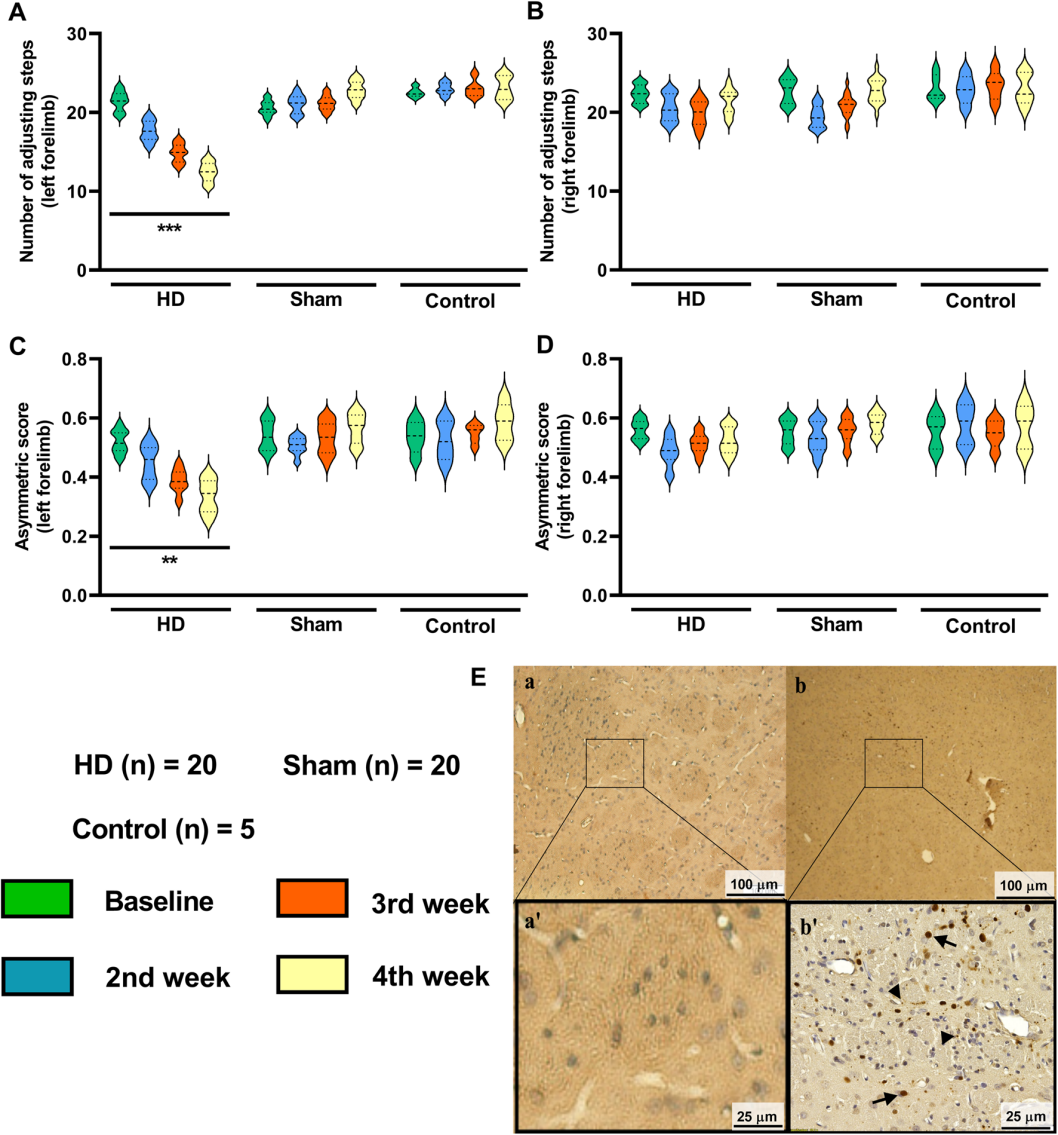
Injection of viral vector pAAV2-CMV-HTT171-82Q-WPRE induces HD model with motor activity deficits. **A**, **B** Stepping test. The HD rats showed a significant decrease in left forelimb activity compared to the sham and control group, but there is no difference in the right forelimb movement. **C**, **D** Cylinder test. The HD rats showed significantly decreased left forelimb asymmetric score compared to the sham and control group at week 6, but no changes were observed in the right forelimb. **p < 0.01; ***p < 0.001 significant difference between the indicated values (ANOVA). **E** EM48 immunohistochemistry staining to detect the Htt aggregations in the rats’ striatum. The presence of Htt aggregations in the AAV2-empty injected area (a, a’) were rarely observed. The aggregations were observed in the AAV2-Htt171-82Q injected area (b, b’)

After injecting viral vector pAAV2-CMV-HTT171-82Q-WPRE in the right CPu of HD animals, significant movement impairment in the left forelimb was found in both stepping and cylinder tests compared to the sham and control group. On stepping test, the affected left forelimb of HD grouped animals exhibited a significant decrease in behavior score from baseline data after six weeks (from 21.44 ± 1.97 to 12.47 ± 2.04; one-way ANOVA, F (3, 76) = 74.75, *p* < 0.001; Fig. 2A). In contrast, behavioral scores of the right forelimb did not show significant changes throughout the six weeks (Fig. 2B).

On the cylinder test, the asymmetric score of the left forelimb (Fig. 2C) also exhibited a significant reduction from baseline data in the HD group over six weeks but not the right forelimb (Fig. 2D). The asymmetric score of left forelimb reduced from 0.53 ± 0.06 to 0.32 ± 0.08 (one-way ANOVA, F (3, 76) = 19.88, *p* < 0.01) (Fig. 2C) in HD animals.

The animals of the sham group and control group did not show any significant difference in their behavior test scores.

### Histopathological change with EM48 aggregations

The mutant Huntington proteins induced aggregations at the infected brain regions that can be detected by EM48 antibody. EM48 positive expression was not observed in the contralateral striatum of AAV2-Htt171-82Q injection (Fig. 2E (a, a')) but it was observed in the ipsilateral striatum of AAV2-Htt171-82Q injection (Fig. 2E (b, b')). The antibody specifically reacts with intranuclear mutant HTT aggregates (black arrow, Fig. 2Eb')) and smaller neuropil aggregates outside the nucleus (black arrow-head). EM48-immunoreactivity was generally detected throughout the nuclei, with a densely stained core resembling the large spherical neuronal intranuclear inclusions typically observed in HD patients or transgenic animals.

### Improvement of motor dysfunctions in HD rats after hESCs transplantation

**Fig. 3:**
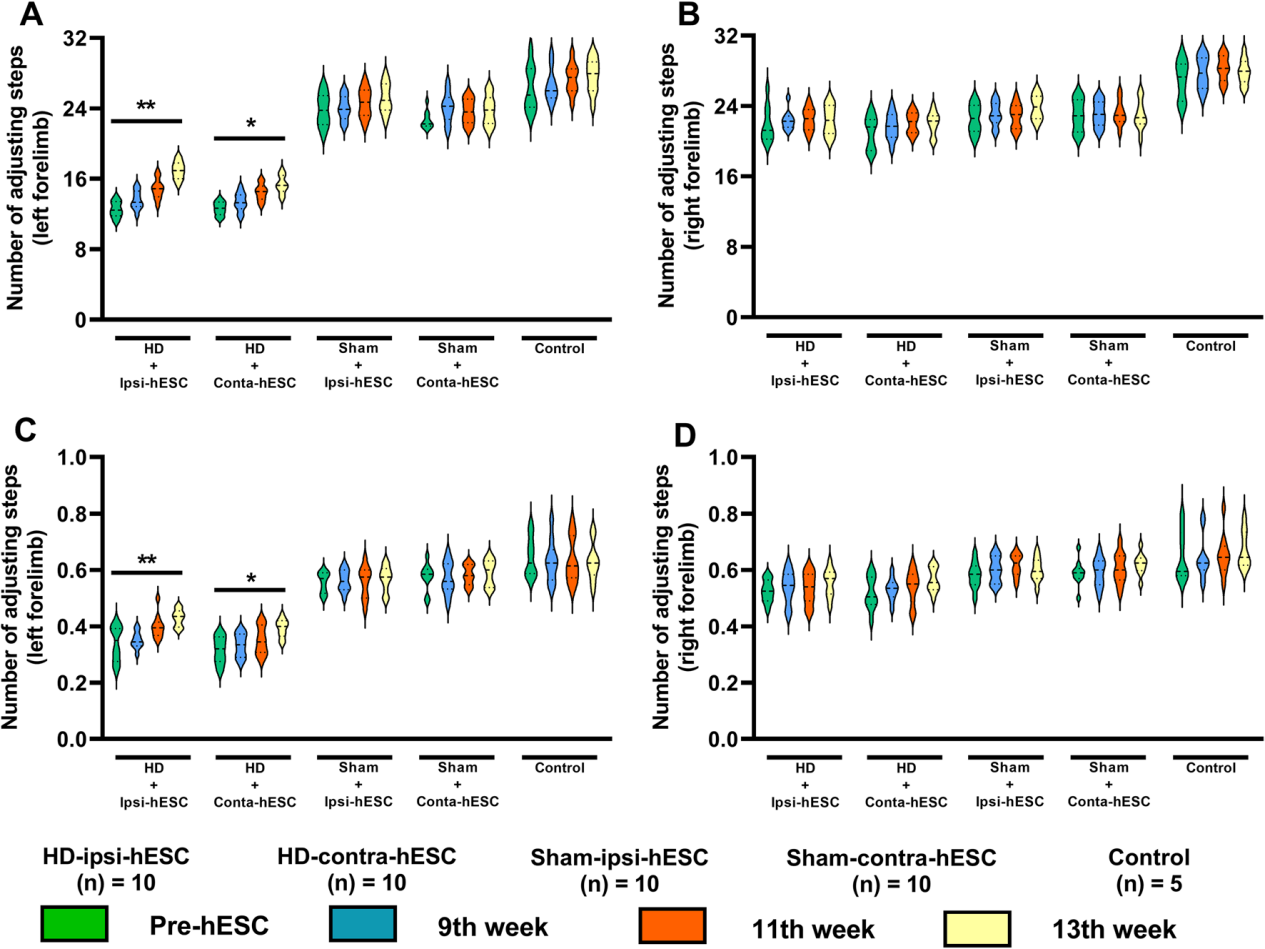
Improvement of motor activities after hESCs transplantation in the HD animals. **A**, **B** The HD rats showed significant improvement in the stepping test in the left forelimb but not in the right forelimb. **C**, **D** The asymmetric score of the left forelimb of HD rats increased significantly after 6 weeks of stem cell transplantation but not in the right forelimb. *p < 0.05; **p < 0.01 significant difference between the indicated values (ANOVA)

After the transplantation of hESCs, the HD rats showed significant improvement in the behavioral score of left forelimb on the stepping test. Animals of HD-ipsi-hESC group showed more significant improvement in their left forelimb (from 12.52 ± 1.44 to 16.96 ± 1.61; one-way ANOVA, F (3, 36) = 13.58, *p* < 0.01) than the animals of HD-contra-hESC group (from 12.61 ± 1.16 to 15.34 ± 1.64; one-way ANOVA, F (3, 36) = 8.83, *p* < 0.05) (Fig. 3A). The transplantation of hESCs did not cause any significant changes in stepping test scores of the right forelimb (Fig. 3B). The asymmetric scores of the left forelimb of HD rats were also found significantly improved after 6 weeks of hESC transplantation. In the animals of HD-ipsi-hESC group, asymmetric score was improved from 0.33 ± 0.08 to 0.43 ± 0.04 (one-way ANOVA, F (3, 36) = 6.501, *p* < 0.01) and in HD-contra-hESC group, asymmetric score gets enhanced from 0.31 ± 0.07 to 0.39 ± 0.05 (one-way ANOVA, F (3, 36) = 4.07, *p* < 0.05) (Fig. 3C). There were no significant changes in asymmetric scores of the right forelimb (Fig. 3D).

Between the two HD groups, the improvement of using left forelimb found in the HD-ipsi-hESC group after six weeks of hESC transplantation was significantly higher than the results found in the HD-contra-hESC group (unpaired t-test (t, df) = (2.229, 18), *p* < 0.05). Sham-ipsi-hESC, Sham-contra-hESC, and control grouped animals did not exhibit any significant changes in their behavior results.

### MRI tracking of SPION labeled embryonic stem cells

**Fig. 4:**
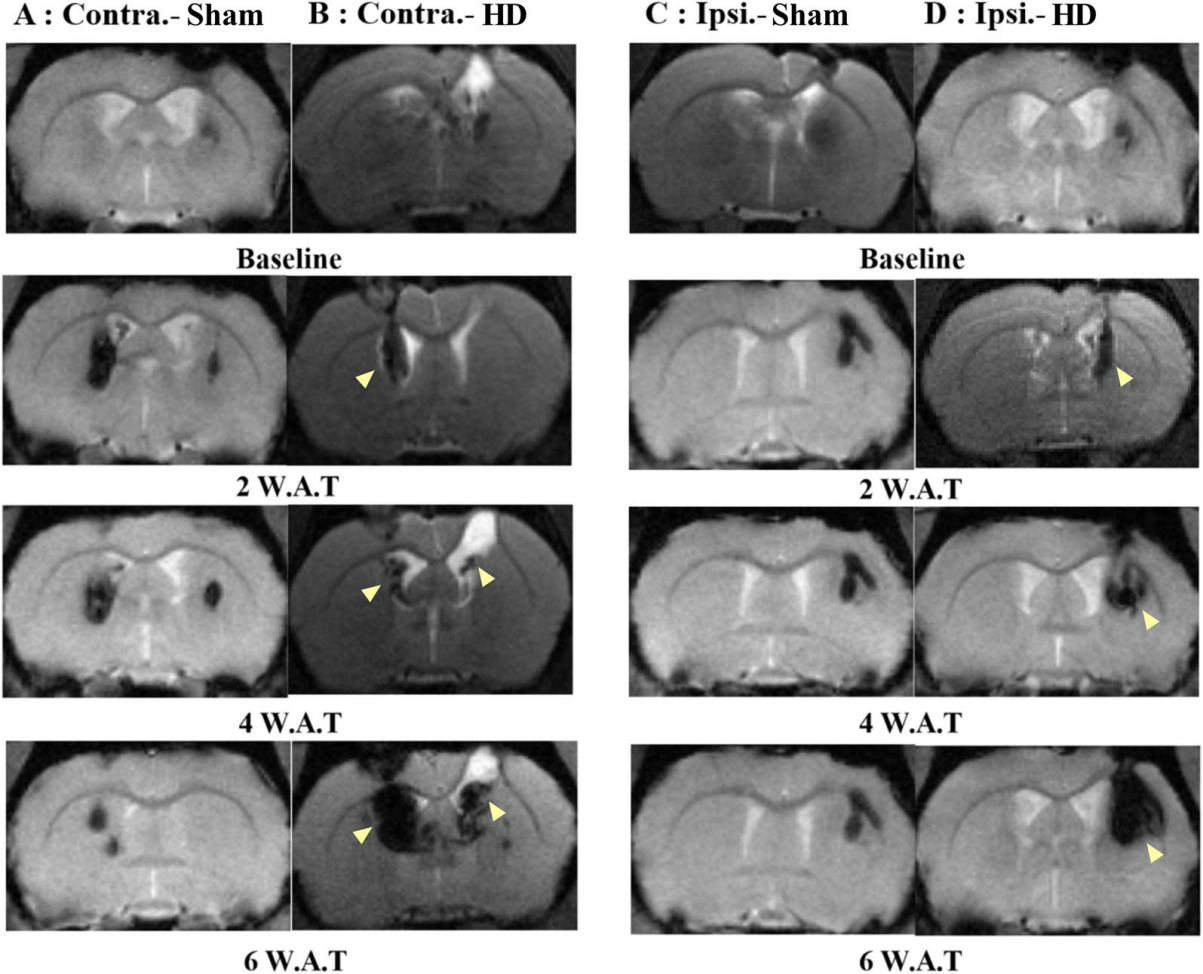
MRI tracking of SPION labeled embryonic stem cells. **A** MRI images of the Sham-contra-hESC group showed a reduction of low signal intensity area in the striatum as time passes and detection of the area in another side of the brain. **B** MRI images of the HD-contra-hESC group showed expansion and migration (yellow arrow) of the low signal intensity area in the striatum as time passes and detection of hESCs in the other side of the brain. **C** MRI images of the Sham-ipsi-hESC group showed no changes of low signal intensity area in the striatum as time passes. **D** MRI images of the HD-ipsi-hESC group showed expansion and migration (yellow arrow) of low signal intensity area in the striatum as time passes

The MRI T2- and T2*-weighted images of the striatum on the coronal plane were taken before and two, four, six weeks after stem cell transplantation for each group, respectively. The trace of the virus injection procedure is shown as a low signal intensity area at the right upper surface of the brain in all the images. SPION labeled stem cells were identified as low signal intensity lesions on the subcortical area. The Huntington group's (Fig. 4B, D) MRI showed the increasing size of low signal intensity area in the striatum where hESCs labeled by SPION were injected. In the animals of the HD-ipsi-hESCs group, the MRI images exhibited the stem cells injection site and its expansion over the weeks in the striatum to recover the destructed neural area (yellow arrow, Fig. 4D). In HD-contra-hESCs animals, the MRI images demonstrated the low signal intensity area in the injected (left) site as well as in the opposite (right) side which expressed the migration of stem cells over the weeks from the injected site (left) towards the degenerated area of the right striatum (yellow arrow, Fig. 4B). These MRI images support the notion of stem cells' prolific potentiality to restore the degenerated cells as well as being able to migrate towards adjacent eroded brain regions. Unlike the HD groups, in the sham group's (Fig. 4A, C) MRI, the low signal intensity area in the striatum showed a reduction (Fig. 4A) in size or no change (Fig. 4C).

The stem cell movement suggested that they survived after transplantation and migrated to an adjacent brain area that was affected by overexpression of mutant Huntington proteins.

### Detection of SPION and production of GABA neurons in the transplanted striatum by hESC-derived neurons

**Fig. 5:**
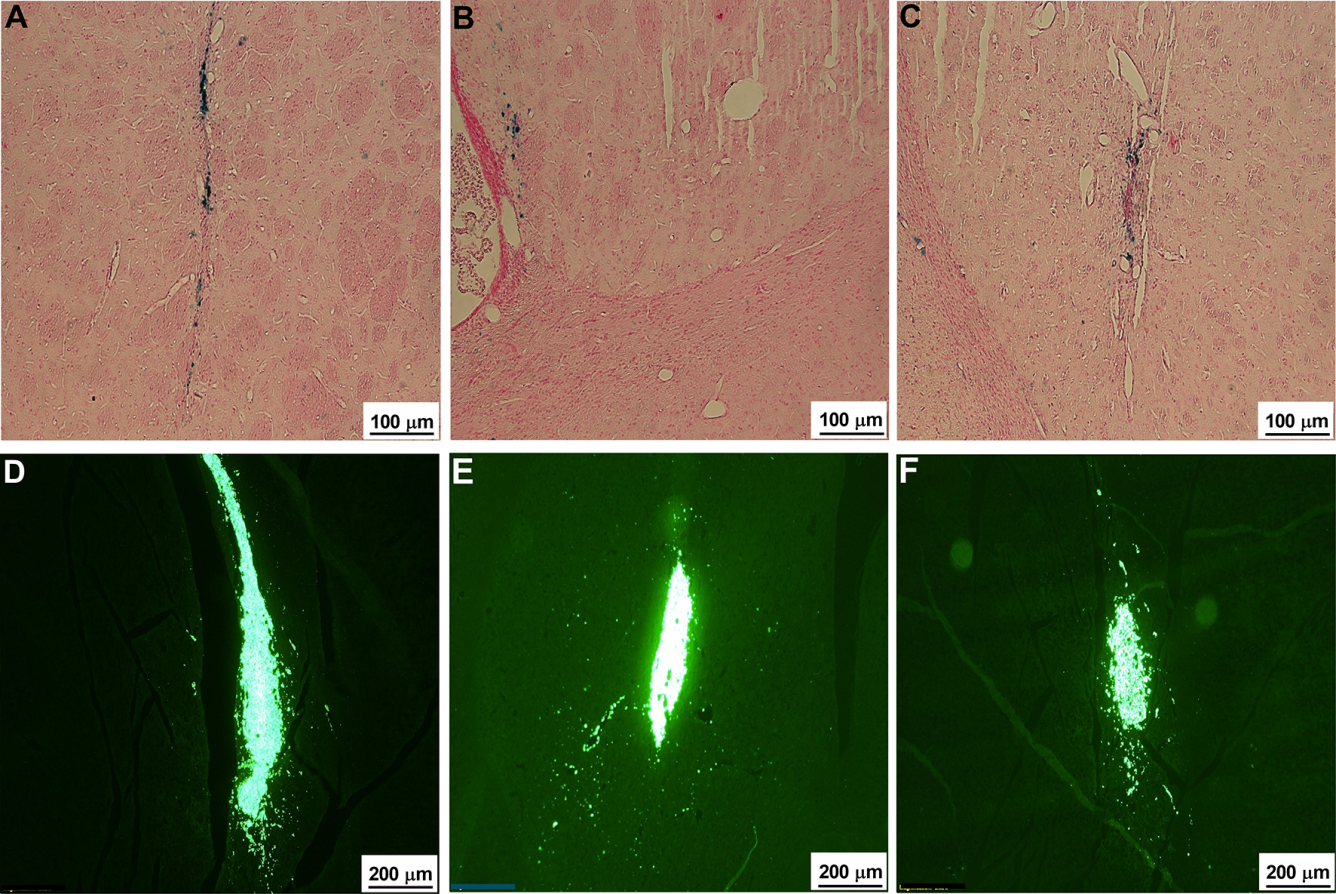
SPION detection and βIII-tubulin, GABA, DARPP32 immunostaining. **A**–**C** The presence of iron was detected by Prussian blue staining. **A**, **B** The appearance of iron in the hESC injected site (**A**) and away from the injected site (**B**). **C** In the contralateral ESCs group, the iron could also be observed in the ipsilateral site. Scale bar: 100 μm. **D**–**F** The transplanted stem cells were positive with βIII-tubulin (**D**), GABA (**E**), and DARPP32 (**F**). Scale bar: **D**–**F** 200 μm

To detect the presence of SPION, we performed Prussian blue staining at one day after transplantation of hESCs and at six weeks after transplantation of hESCS. The presence of SPION was seen in the injected site (Fig. 5A) and after six weeks, far from the injected site (Fig. 5B). In case of hESCs that were transplanted contralaterally to the virus injection site, SPION was also detected at the site of virus injection after six weeks (Fig. 5C). To determine if the transplanted hESC derived into GABA neurons, we also obtained immunostaining for βIII-tubulin, GABA, and DARPP32. The stem cells were strongly positive with βIII-tubulin (Fig. 5D) at the injected area, and the derived neurons were also positive with GABA (Fig. 5E) and DARPP32 (Fig. 5F).

### hESCs transplantation effects on glia and neurons

**Fig. 6:**
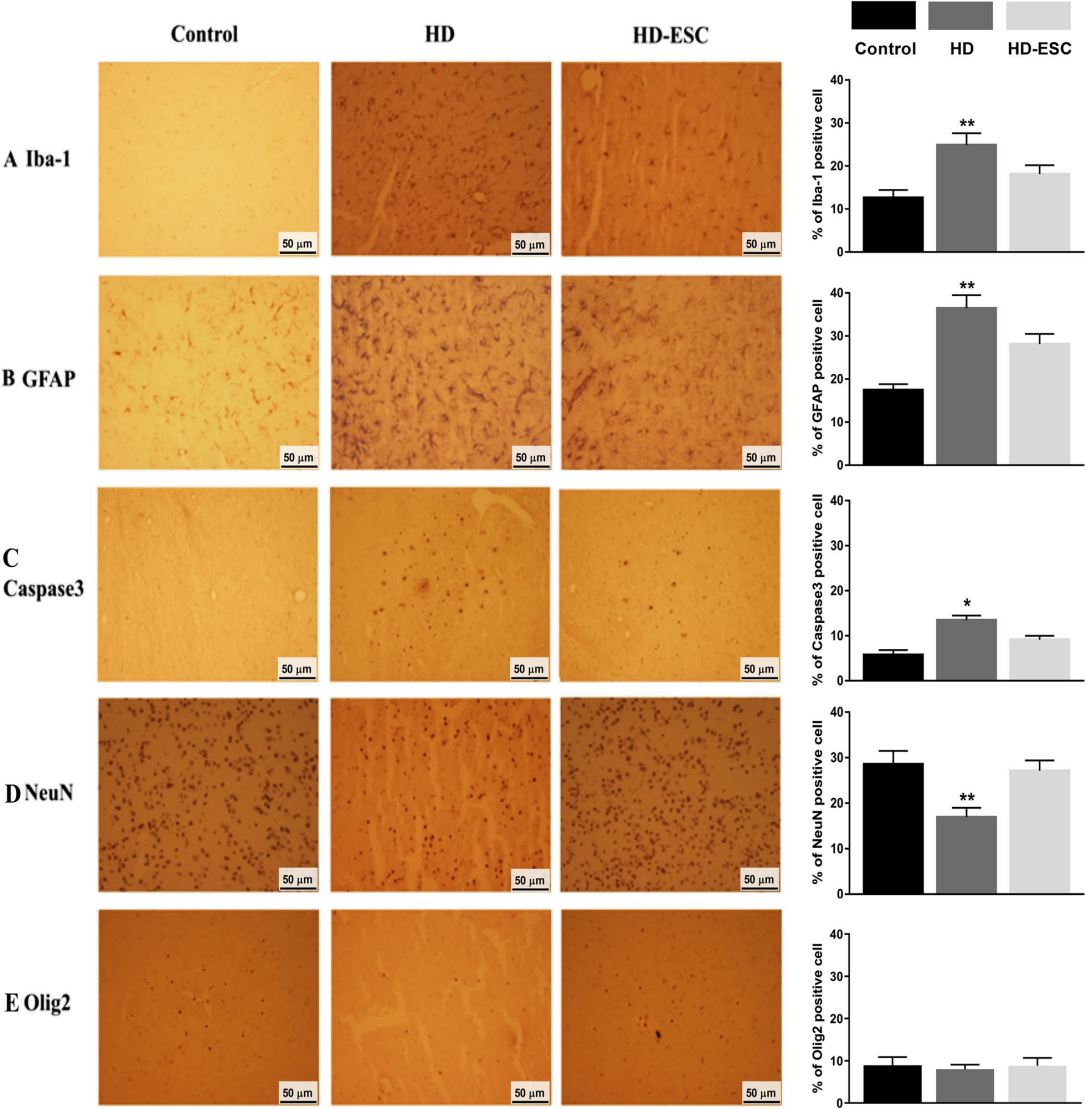
Immunohistochemistry and semi-quantification results showing human embryonic stem cell transfection reverses the effect of AAV2-Htt171-82Q on microglia, astrocytes, and neurons. **A**, **B** The microglia and astrocytes were strongly activated by the infection of AAV2-Htt171-82Q (HD group) but less activated after cell transplantation (HD-ESC group). **C** The expression of caspase marker was increased in HD cells but get decreased after stem cell transplantation. **D** The neuronal marker (NeuN) was strongly expressed after cell transplantation compared with the HD group. **E** Oligodendrocytes marker expression in control, HD, and stem cell transplanted cell. *p < 0.05; **p < 0.01 significant difference between the indicated values (ANOVA)

The transfection of stem cells reversed the effect of AAV2-Htt171-82Q in the HD rat's striatum (Fig. 6). Rat brains of the HD group showed the activation of microglia (Iba-1 positive, Fig. 6A), astrocytes (GFAP positive, Fig. 6B), and apoptosis marker (Caspase3 positive, 6C) after viral vector injection. The expression of Iba-1, GFAP, Caspase3 were lower after stem cell transfection in the HD-hESC group and semi-quantification results also revealed that Iba-1, GFAP, and Caspase3 activation were higher in HD cells than control and HD-hESC cell. Both IHC and semi-quantification results showed that the neuronal marker (NeuN, Fig. 6D) in the stem cell transfected groups was higher than in the HD group. Staining for the oligodendrocyte marker (Olig2, Fig. 6E) did not exhibit a significant difference between the control, HD group, and stem cell transfected group.

## Discussion

We have shown that hESCs could migrate to degenerated brain areas and alleviate motor function in AAV2 Htt171-82Q transfected Huntington disease rat model. Cell tracking was conducted using SPIONs for labeling and detected by 4.7 T MRI. The model was generated by injecting a viral vector that carries the mHtt gene directly into the rat striatum. The aggregations induced by mutant Htt proteins were detected by EM48 immunohistochemistry staining. After stem cell transplantation, transfected rats showed significant improvement in motor function that could be observed in both contralateral and ipsilateral ESCs transplantation groups.

The findings from animal model studies have helped to elucidate essential pathways that are disrupted in HD and have provided valuable insights into the pathogenesis of this disease [30]. Several animal models including drug-induced rat models using Quinolinic and kainic acid, transgenic and knock-in models and viral vector delivery mHtt gene directly to animal brains were developed [31–37]. The Htt171-82Q vector delivered by lentiviral transfected to the animal putamen had successfully generated abnormal movements that resembled typical motor deficits of HD [35, 37]. However, lentiviral vectors have some disadvantages such as unpredictable loci of viral progeny insertion and the induction of immune responses [38]. Because AAV induces a weaker immune response compared to lentiviral vectors, it can be more safely introduced into primates [37]. In our previous study, we showed that using AAV2 for introducing the human mHtt gene to the rat brain could induce a safer and more stable animal model of Huntington’s disease compared to the toxic model [25]. AAV2 primarily attaches with “Heparan Sulfate Proteoglycan (HSPG)” and it is well established that HSPG centrally participates in the protein aggregation process and it can facilitate inflammatory reactions by stimulating the production and release of the inflammatory cytokines in chronic inflammatory conditions in different neurodegenerative diseases [39, 40]. In the case of HD, the severe loss of GABAergic medium spiny neurons in the striatum was targeted as the first character of the HD brain and the degeneration starts in the caudate and putamen. And due to this striatal tissue death, the natural motor activity of an entity face hindrances as the important role of the striatum in controlling the movement of the opposite side of the body is irrefutable [41–43]. Behavior results of this study also exhibited the same phenomenon with significant motor activity deficits in the left forelimb after injecting the Htt virus in the right striatum.

In our study, we used the magnetic resonance imaging (MRI) technique with superparamagnetic iron oxide nanoparticles (SPIONs) for tracking human embryonic stem cells in a transfected Huntington rat model. MRI is a non-invasive imaging technology that produces three-dimensional detailed anatomical images without the use of damaging radiation. The technique has been applied broadly in medicine, with the help of contrast agents such as Gadolinium and superparamagnetic iron oxide (SPIO) in improving the visibility of cells or tissues. SPIO particles have no adverse effects on the viability or proliferation of labeled cells [44, 45]. Therefore, they are promising candidates for labeling stem cells. Previous studies demonstrated SPION labeling in a variety of human [46, 47] and animal [48, 49] stem cells. Moraes et al. (2012) indicated SPION-labeled MSC as a potential tool for MRI tracking and confirmed that internalized SPION induced sufficient cell MRI contrast to allow in vivo tracking of transplanted MSC [23]. They observed a visible and local hypointense MRI signal at the transplanted area one hour, 1, 7, 14, 28, 42, and 60 days after transplantation, with the migration of cells, appeared after 7 days of transplantation. We also observed similar outcomes in MRI after transplanting hESC into the HD rat striatum that supported the notion that SPION-labeled hESC also is a prospective tool for MRI tracking. Besides, our MRI results were not showing iron artifact caused by hemorrhage because it is known that the hemorrhage signal changes as time passes. As we used T2 and T2* weighted images and our images were taken between the second to sixth weeks after hESC transplantation, had the lesion been caused by hemorrhage it would have been of high signal intensity (bright). But our images showed low signal intensity lesions. In hemorrhage, T2 and T2* weighted imaging get affected by the hemoglobin oxygenation state and presence of cell lysis. While containing RBC, both deoxyhemoglobin and methemoglobin result in loss of signal during cell lysis because of their uneven distribution of paramagnetic effects. After that, the faded signal comes again due to unevenly distributed paramagnetic effects of hemosiderin and ferritin ingested by monocytes and macrophages. However, our images exhibited a consistent low signal throughout the observation period, and moving to the other side is not possible for hemorrhage. Therefore, we can consider our images showing a low signal of SPION [50, 51].

We chose MRI as a cellular tracking method because of its ability to detect single transplanted cells in vivo and its high resolution that can be translated to big animals and humans [52–54]. Other tracking methods that are using in vivo are bioluminescence imaging (BLI), positron emission tomography (PET) – computed tomography (CT) scans, and fluorescence imaging (FLI) with quantum dots (QD). However, to date, these methods could not be applied for long-term tracking in large animals. BLI is not sensitive as MRI and can cause immunogenicity, high absorbance, and scattering of luminescence that it would be limited in small animal [55]. PET-CT scans require repeated injections of radioactive material which can lead to damage to surrounding normal tissues [56]. FLI with QD also causes light scattering and unknown long-term biological health effects [54]. Compared to them, MRI still is the most proper long-term cell tracking method at this moment.

Stem cell therapy has been applied in research of neurodegenerative diseases such as Parkinson’s and Huntington’s disease. In HD, researchers have used neural stem cells (NSCs), mesenchymal stem cells (MSCs), and induced pluripotent stem cells (iPSCs) as a treatment in vivo*.* These types of stem cells showed improvement in motor functions in the animal models [17, 57–59]. However, in the case of hESC, their pluripotency allows them to differentiate into neuronal progenitor cells and all three germ layers in vitro [60–62] and in vivo [63, 64], and that is the reason of aroused tremendous research interest regarding their application as one of the promising approaches for the treatment of neurodegenerative diseases. Recent studies have revealed that hESC cultures with Matrigel improved behavioral outcomes as well as differentiated into neuronal cells primarily [65] and our hESCs were maintained with Matrigel-coated plates. ESC demonstrates long-term stability which favors them to integrate into the introduced structure. ESC possesses several transcription factors such as OCT3/4, NANOG, and SOX2, as well as neural derivatives with the capability of secreting various neurotrophic factors, to provide trophic clinch in the damaged area. Besides, ESC-derived neurons take part in the reconstruction of cortical circuitry of the similar areal identity [66]. The ability of differentiation into striatum GABAergic neurons from human and mouse embryonic stem cells in rodent HD models, as well as, migration of stem cells towards neural degenerated area have been reported earlier in several studies [3, 67–70], and the derived GABAergic neurons connected with endogenous cell and corrected motor deficits in the model [3]. We also observed the migration of the stem cells towards the damaged area in our HD group with both ipsilaterally and contralaterally transplanted conditions and our behavior test results showed improved motor activities after hESCs transplantation in HD rats. We believe that another important aspect of hESC’s successful proliferation and migration in our study was that to differentiate into area-specific neuronal progenitor cells in the host body, grafted cells have to survive and overcome the host versus graft reaction, and we transplanted the hESCs in an AAV-HTT171-82Q injected HD rat model. Transplanted cells into transgenic animal models demonstrated lower cell survival because of the ongoing progressive pathology. Moreover, in the transgenic line, there remains a densely packed graft core due to the absence of a lesion area, which might produce impediments in survival, migration, and integration of grafted cells. And it has already been revealed that extensive neuronal cell death is the prerequisite for the survival and integration of transplanted cells and acute and delayed immune-rejection phases start at no earlier than 4 weeks post-transplantation [71, 72]. In our study, animals were transplanted with hESC both ipsilaterally and contralaterally to the striatal lesion site. The stem cells were found positive with βIII-tubulin (early neuronal marker), GABA, and DARPP32, which suggested that they were derived into GABAergic medium spiny neurons at the transplanted striatum. Therefore, our study results showed that hESCs survived and replaced the degenerated striatal neurons and reinstate the motor activity in an AAV2-HTT171-82Q HD rat model.

There were some limitations in our study. We only checked the effect of hESCs in improving the motor skill of the model with transplantation of the stem cells at a concentration of 1.25 × 10^5^ cells/μl (5 μl per rat). We neither compare SPION with other contrast agents nor MRI with other tracking methods. We did not inject any immunosuppressant agent. Further studies with a larger sample size and longer period could be generated to find more evidence of the effect of human ESCs in other behavior tests in HD animal models.

## Conclusion

To recapitulate, our study showed that the transplantation of human embryonic stem cells could migrate and ameliorate motor function in an HD rat model. By using MRI, we detected the migration of SPION-labeled stem cells at the lesion area as well as the contralateral hemisphere. This study provides potential data and hypotheses which can be applied for further studies with human embryonic stem cell transplantation therapy for HD studies and other neurodegenerative disorders.

## Data Availability

The datasets used and/or analyzed during the current study are available from the corresponding author on reasonable request.

## Abbreviations

HD: Huntington’s disease
mHtt: Mutant huntingtin protein
hESCs: Human embryonic stem cells
MRI: Magnetic resonance imaging
SPIONs: Superparamagnetic iron oxide nanoparticles
Ipsi: Ipsilateral
Contra: Contralateral
CPu: Caudal putamen
